# Navigating Ghana's economic waters: Exploring the impact of Fiscal and Monetary policies on stock market performance

**DOI:** 10.1016/j.heliyon.2024.e38761

**Published:** 2024-10-09

**Authors:** Benjamin Blandful Cobbinah, Yang Wen, Francis Atta Sarpong

**Affiliations:** a College of Economics, Shenzhen University, Shenzhen, China; b School of Finance, Zhongnan University of Economics and Law, Wuhan, China

**Keywords:** Fiscal Policy, Monetary Policy, Stock market performance, EGARCH, ARDL

## Abstract

This study leverages the Autoregressive Distributed Lag (ARDL) and Exponential Generalized Conditional Heteroscedasticity (EGARCH) models to conduct a thorough examination of the impact of fiscal and monetary policies on the Ghanaian stock market from 1990 to 2022. Key findings indicate that government spending and tax revenue, as components of fiscal policy, are positively associated with stock returns, contrasting with the negative influence of the industrial production index. On the monetary policy front, interest rates are found to negatively affect stock performance, while exchange rates and the money supply exert positive influences. In the short term, government spending enhances stock returns, although the effects of GDP and the industrial production index are inconsistent, with exchange rates and money supply demonstrating a negative impact. The study underscores the profound sway that policy decisions have on stock market dynamics, underscoring an urgent need for investors and policymakers to closely monitor policy shifts and their market reverberations. A pivotal policy recommendation emerging from this research is the strategic synchronization of fiscal and monetary policies by policymakers to underpin stock market stability and growth. Such harmonization can counteract the adverse effects of policy-induced volatility, cultivating an investment-friendly climate. Investors and policymakers are encouraged to draw upon a spectrum of credible sources, encompassing financial news, governmental releases, and market analyses, to remain abreast of policy evolutions. This research offers precious perspectives on the nexus between economic policies and market movements, offering value for academic inquiry and informing practical decision-making strategies.

## 1 Introduction

Economic shocks necessitate the implementation of counteractive fiscal and monetary policies to ensure stability. Fiscal policy, a component of macroeconomic regulation, operates through the strategic adjustment of taxation and public expenditure [1]. This approach serves as a countercyclical tool, aiming to mitigate the impacts of economic downturns and enhance societal welfare. Monetary policy, on the other hand, is conducted by central banks, which utilize interest rates and control over the money supply as primary levers [1]. The Bank of Ghana exemplifies this by targeting monetary policy to stabilize exchange rates, achieve predetermined inflation objectives, and stimulate economic growth [2]. To realize these macroeconomic goals, policymakers employ diverse transmission mechanisms, influencing economic activity across various sectors [2]. Fiscal policy, often referred to as countercyclical demand management, involves modifications to government revenue collection and expenditure strategies [3]. These modifications are designed to influence aggregate demand and, consequently, the overall level of economic activity.

The efficacy of monetary policy in influencing economic stability remains a contentious issue, particularly in the context of African economies. Despite numerous policy adjustments, many African nations continue to grapple with economic challenges [4]. This scenario underscores the critical role of stock markets in driving trade and industrial growth, which in turn significantly impact the broader economy. Investors, recognizing the pivotal influence of stock markets, meticulously assess the performance of composite market indices prior to making investment decisions [5].

Investment in the stock market is a strategic avenue for potential future returns, with dividends and capital gains being the primary incentives [6]. However, the inherent risks, uncertainties, and financial instabilities associated with stock markets pose a threat to investors' capital. The preference of foreign and state investors for US assets further highlights the interconnectedness of global financial markets. Economic turbulence or market perils in the US could deter investment, potentially leading to a significant devaluation of the dollar and consequential damage to the nation's political and economic standing [7].

The US market, being the largest financial market globally, exerts considerable influence on other major stock markets and international economic conditions [7]. Empirical research on the interplay between macroeconomic variables and stock market behavior is categorized into two primary streams [8]. The first stream investigates the initial correlation, utilizing various methodologies such as Vector Autoregression (VAR), multivariate cointegration, and Vector Error Correction (VEC) models to establish a robust empirical link. The second stream, building upon the first, delves into the relationship between stock market characteristics and the risk and volatility of macroeconomic aggregates. This research is predicated on the assumption that the volatility of macroeconomic variables significantly complicates stock market planning and execution [9].

**Fig. 1:**
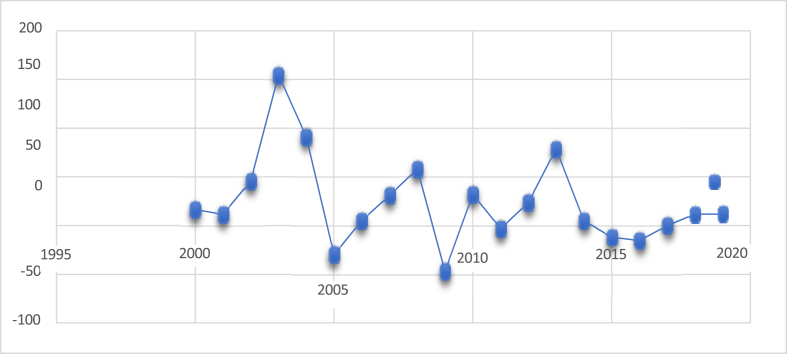
GSE Composite Index 2000 To 2020

The Ghana Stock Exchange (GSE) has been recognized as one of the top-performing exchanges globally, with historical data attesting to its remarkable capital appreciation. In 1993, the GSE achieved a capital increase of 116%, ranking it as the sixth best-performing developing stock market [10]. Further analysis by Birinyi Associates in 1994 confirmed the GSE's leading position among developing markets, with an impressive 124.3% growth at the index level [11]. Despite fluctuations post-1995 attributed to internal shocks and macroeconomic instability, the GSE demonstrated resilience as illustrated in Fig. 1. In 2003, it was distinguished by the Morgan Stanley Capital International Global Index as the world's highest-performing market, with an annual return of 154.7% in Ghanaian cedis or 144% in US dollars, significantly surpassing the global average of 30% [11]. This exceptional performance has garnered considerable attention from researchers and investors, highlighting the GSE's potential despite its relatively nascent status compared to other African stock exchanges.

**Fig. 2:**
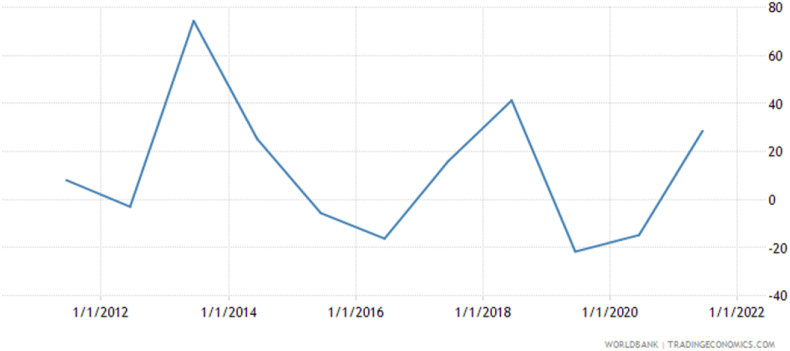
Ghana stock market trend

Source: World Bank trading economics

This study delves into the intricate relationship between fiscal and monetary policies and their influence on stock market returns, with a particular focus on the Ghanaian context. The 1980s marked a significant transformation in Ghana's financial sector with the introduction of the Structural and Economic Recovery Programmes, which catalyzed a series of economic reforms. These included the adoption of a flexible exchange rate system, deregulation of interest rates, and the implementation of credit rationing policies, contributing to the maturation of Ghana's financial landscape over the subsequent decades. Our decision to focus on Ghana is underpinned by multiple factors. Since achieving independence from British colonial rule in 1957, Ghana has employed a coordinated approach to monetary and fiscal policy as a means of economic governance. However, the existing literature on the impact of these policies on the stock market is predominantly centered on developed economies, with emerging economies like Ghana receiving scant attention. This research aims to contribute to the literature by examining the interaction of fiscal and monetary policies on the stock market within the timeframe of 1990 to 2022. The World Bank's development indicators reveal a noteworthy statistic for Ghana's stock market performance; the return for the year 2021 was recorded at an impressive 28.63% as seen in Fig. 2. This data, along with current figures, historical trends, forecasts, and predictive models, was sourced from the World Bank in September 2023, providing a robust foundation for our analysis.

This study aims to address several research questions pertinent to the intersection of fiscal and monetary policies with stock market performance in Ghana. Specifically, the investigation will explore the relationship between the country's Composite Index (CI), a key metric of stock market performance, and the components of monetary policy, including the interest rate (INT), money supply (MS), consumer price index (CPI), and exchange rate (EXC). Additionally, the study will examine the interplay between fiscal policy indicators—such as GDP, government expenditure, tax revenue, and the industrial production index (IPI)—and stock returns as measured by the CI. A central theme of this research is the impact of macroeconomic policy volatility on stock market planning and outcomes, a factor known to introduce significant challenges in financial forecasting and decision-making. The study will, therefore, reassess the potential effects of fiscal and monetary policy volatility on the Ghanaian stock market. Furthermore, it will investigate the presence of any significant interactions or spill-over effects of these policies on stock returns. To empirically analyze the volatility clustering inherent in financial time series data, the study will employ the Exponential Generalized Autoregressive Conditional Heteroscedasticity (EGARCH) model. This model is well-suited for examining the impact of policy variations on stock market volatility and returns. Complementing the EGARCH approach, the Autoregressive Distributed Lag (ARDL) model will be utilized, offering a robust framework for analyzing time series data where observations are correlated with their past values.The structure of this research is methodically organized into sections that include a review of related literature, a detailed methodology, results, discussion, and conclusions, ensuring a comprehensive exploration of the research questions at hand.

## 2 Review of Related Studies

### 2.1 Theoretical Underpinning

Renowned researcher Tobin crafted a model illustrating the potential repercussions of fiscal-monetary policy shifts on stock market returns. His work underscored the significant influence that both monetary expansion and fiscal deficits exert on stock market performance. Recognizing the impact of government actions on forthcoming monetary policy decisions, Tobin's insights [[12], [13]] further highlighted the profound effects of monetary growth and fiscal imbalances on stock market dynamics. The intricate relationship between fiscal policy and stock market behavior has been dissected through various theoretical lenses, revealing a multifaceted interplay.

### 2.2 Theoretical note on Fiscal Policy and stock market return

Traditional Keynesian macroeconomics, grounded in the IS-LM model, predominantly emphasizes the role of aggregate demand in shaping short-term economic activities, while the supply side often receives less attention [14]. The theory suggests that significant budget deficits have the potential to raise interest rates, thereby attracting foreign investment [15]. Additionally, it posits that tax cuts or financial expansions funded by the public sector could deplete private savings by increasing disposable income, which in turn fuels consumption—a notion that aligns with Keynesian fiscal policy. This policy framework scrutinizes the secondary effects of fiscal deficits on savings and investments. The dynamics between a savings deficit, investment, and current account balances are contingent upon the openness of a nation's capital markets, which may subsequently influence stock returns [14].

The economic theory of rational expectations, which emerged in the 1970s, posits that individual—encompassing consumers to investors—base their future economic expectations on all available information [16]. This theory presupposes a high degree of economic literacy and awareness among market participants, which should, in theory, lead to more precise predictions of economic fluctuations and a reduction in their inherent unpredictability. The rational expectations paradigm is instrumental in the formulation and assessment of economic policies. Nonetheless, critics contend that the assumptions of perfect information access and complete comprehension are overly optimistic and may result in divergences from the theory's predictions. A thorough understanding of this perspective is vital for policymakers to devise effective economic strategies [16].

The Ricardian Equivalence Theorem (RET) is a cornerstone of macroeconomic theory, proposing that fiscal stimulus financed by government deficits may not invigorate the economy as expected [17]. RET contends that increased public spending or tax cuts, when debt-financed, could result in reduced private consumption, thus undermining the potency of fiscal policy [18]. This occurs as individuals anticipate future tax hikes to service the debt, prompting them to save more and consume less. Scholars [9] propose that the Ricardian neutrality hypothesis offers a sophisticated explanation for the muted effects of fiscal policy on the real and financial sectors, particularly in the absence of complementary monetary policy measures. Conversely, other researchers [19] question the plausibility of the theory's assumptions, arguing that rational individuals would anticipate the future tax liabilities associated with government debt, which could lead to higher savings and lower spending, ultimately affecting stock market returns.

The ongoing discourse surrounding traditional Keynesian economics, rational expectations, and the Ricardian Equivalence Theorem underscores the complexity and evolving nature of macroeconomic theory. With the global economy continually facing new challenges and transformations, the need for robust, relevant, and nuanced research is more critical than ever. Further studies are essential to refine our understanding of how fiscal and monetary policies interact within different economic contexts and to what extent they influence market behaviors and outcomes. Continued research is particularly necessary to reconcile theoretical predictions with real-world observations, taking into account the limitations and assumptions of each economic model. By interrogating and expanding upon existing theories, economists can offer more accurate guidance to policymakers and better navigate the complexities of modern economic challenges. This pursuit of knowledge is indispensable for fostering sustainable economic growth, stability, and resilience in an increasingly interconnected world.

### 2.3 Theoretical note on Monetary Policy and stock market return

Monetary policy is designed to achieve macroeconomic stability, foster growth, and enhance societal welfare. It is intricately linked to the stock market through two primary channels: the stock market's reflection of expectations about future macroeconomic fundamentals to monetary authorities, and the direct impact of monetary policy shocks on stock market behavior. This study delineates several mechanisms by which monetary policy influences the stock market, including interest rate adjustments, portfolio balance effects, credit availability, discount rates, and valuation channels.

The interest rate channel, a cornerstone of Keynesian theory, is the primary conduit through which monetary policy signals are transmitted to the economy [9]. Defined as the cost of deferring consumption or the return on capital [20], interest rates play a pivotal role in determining the present value of future cash flows. An increase in interest rates can precipitate a decline in stock values by reducing these present values. Thus, a balanced interest rate policy that maintains an equitable financing cost is essential for sustainable stock market development [20]. Bernanke's analysis of the Federal Reserve's 2010 policy highlights the Portfolio Balance Channel's role in redirecting investment from government securities to higher-yielding corporate instruments, thereby stimulating the economy [21]. This channel, complemented by the credit flow channel, increases the money supply and potentially boosts stock prices.

Monetary policy's manipulation of asset yields prompts investors to reassess their portfolios, often leading to a shift from lower-yielding bonds to riskier assets like stocks during expansionary policies [22]. This behavior underscores the nuanced relationship between monetary policy and investor decision-making. A profound understanding of how monetary policy shapes the interbank market and, by extension, the broader economy is essential. The bank credit channel, a critical component of monetary policy transmission mechanisms (MPTMs), is particularly influential in economies with financial frictions [[23], [24], [25]]. By adjusting the cost of credit, monetary authorities can directly affect investment levels and, consequently, stock market dynamics. The market value of a company, derived from the present value of its future cash flows, is sensitive to these investment fluctuations [9]. The discount rate, central to valuing future cash flows, reflects the time value of money. When central banks adjust the discount rate, it can reverberate through the stock market and the wider economy, particularly in industries with enduring market leadership [26]. This rate is the lifeblood of bank liquidity management and is instrumental in the central bank's broader economic strategy.

### 2.4 Empirical Evidence on the subject of Fiscal-Monetary Policies on the Stock market

A study by [27] delves into the semi-strong form of the market efficiency hypothesis, with a particular focus on the Romanian stock market and its reaction to fiscal policy information. Using the ADR-bound testing methodology, the research, initially developed in 2001, examines the extent to which stock prices reflect information about fiscal policies. The findings support the view that stock prices comprehensively incorporate past fiscal policies but reveal a nuanced response to unexpected fiscal policy news, which has an immediate effect on stock returns, unlike the delayed impact of anticipated fiscal policy information. Additionally, the study suggests that the integration of monetary policy information into stock prices is inefficient, with its influence on stock returns being more pronounced in both the short and long term [[27], [28]].

Shifting focus to the Jordanian Amman Stock Exchange, a study by [29] explores the effects of fiscal and monetary policies on stock returns from 2006 to 2016. Through a descriptive-analytical approach and multiple regression analysis, the research uncovers a significant impact of inflation on stock returns, establishing a causal link between stock returns, interest rates, and inflation. The study also finds a long-term cointegration relationship among all variables, underscoring the need for policymakers to consider the interplay between monetary and fiscal policies when formulating government policies, given their potential effects on stock returns [29].

[30] evaluates the market efficiency hypothesis using fiscal policy data from the New York Stock Exchange (NYSE). Employing the Autoregressive Distributed Lag (ARDL) bounds testing approach on data from 2008 to 2018, the research indicates a high degree of market efficiency, as stock prices fully incorporate fiscal policy data and prior actions. However, the NYSE stock index shows distinct reactions to the most unexpected fiscal policy updates in the immediate period [30]. The study also explores the correlation between stock values and the prices of consumer goods, manufacturing, and oil, revealing that while rising inflation or consumer prices may enhance business profitability, increases in oil prices tend to negatively affect stock values, suggesting that understanding these relationships can improve stock market efficiency [30].

[31] investigates the impact of unexpected changes in the European Central Bank's (ECB) monetary policy on the returns of German surplus stocks. Using an event study approach and the Vector Autoregression (VAR) framework, the study assesses the influence of both traditional and non-conventional monetary policies on stock returns. The analysis reveals that changes in anticipated dividend payments significantly drive fluctuations in German stock returns that exceed expected levels, and the current interest rate system significantly influences the stock market's reaction to monetary policy shocks [31].

Extending the investigation to the Vietnamese financial market, a study by [32] examines the impact of monetary policy on stock prices from 2006 to 2015. Utilizing a linear regression model and analyzing key monetary indicators alongside stock prices, the research, which applies the GJR-GARCH and ARDL models, uncovers a statistically significant and enduring negative relationship between monetary factors and stock prices in Vietnam [32].

[33] also explores the transmission of policy effects on economic growth and inflation in the BRICS nations, focusing on the interaction between exchange rates, monetary policy, fiscal policy, and external balances. The panel vector autoregression (VAR) analysis results indicate that while fiscal policy shocks have a relatively minor impact, monetary policy shocks significantly influence actual economic activity. The findings are consistent with economic research on the Italian economy, highlighting the interplay between the inflation and interest rate channels and their influence on stock market behavior [34].

Recent empirical studies on international spillovers have employed diverse methodologies, including the global VAR approach by [35,36], the two-phase GARCH-in-mean model by [37], and the multi-country structural dynamic factor model by [38]. The panel technique, as used by [[39], [40]], demonstrates that changes in US monetary policy have a negative impact on the stock values and exchange rates of fourteen emerging market economies [[39], [40]]. Furthermore, a study by [41] investigates the role of exchange rate regimes in the transmission of monetary policy effects between advanced and emerging nations, finding that economies with time-varying spillover and a flexible exchange rate regime are better shielded from global shocks [41].

The diverse methodologies and findings from studies examining the impact of fiscal and monetary policies on stock markets underscore the complexity of global financial systems. The nuanced effects of policy changes on market efficiency, as seen in the Romanian, Jordanian, Vietnamese, and German contexts, highlight the need for a deeper understanding of these dynamics. The varying responses to fiscal and monetary stimuli across different economies, particularly the BRICS nations, further emphasize the importance of tailored policy approaches that consider national and regional particularities. Moreover, the examination of international spillovers and the role of exchange rate regimes in policy transmission reveals the interconnectedness of global financial markets. As economies continue to grapple with the challenges posed by globalization, including the management of inflation, interest rates, and external balances, the need for robust empirical research becomes increasingly vital. Such studies not only contribute to the theoretical understanding of economic principles but also provide actionable insights for policymakers. By illuminating the mechanisms through which fiscal and monetary policies influence stock markets and economic growth, these investigations aid in the formulation of more effective and responsive economic strategies. In an era marked by economic volatility and rapid policy shifts, the pursuit of knowledge in this domain is indispensable for navigating the intricacies of global finance and ensuring economic stability and prosperity.

## 3 Materials and Methods

### 3.1 Variable Measurement

For the study to remain accurate and consistent, different definitions were essential. Among the crucial factors examined were:

#### 3.1.1 Dependent variable: Stock Market Returns:

This study employs the composite stock index, a critical metric for assessing the overall level of stock market prices [42]. To explore the correlation of interest, the research utilizes a comprehensive dataset spanning from 1990 to 2022. The dataset comprises real data on the relevant variables, providing a robust foundation for the analysis. The primary source of the data is the annual statistical pamphlets published by the Central Bank of Ghana. These pamphlets, made available on the Central Bank's official website, offer a reliable and authoritative source of economic and financial data for the country. By leveraging this data, the study aims to provide a thorough and evidence-based examination of the stock market's performance over the specified period.

#### 3.1.2 Independent Variables: Fiscal Policy and Monetary Policy

This study anchors its analysis on fiscal policy and monetary policy, which are the primary independent variables under investigation, as detailed in Table 1. Fiscal policy is approximated through indicators such as the industrial production index (IPI), government spending (GS), government revenue (TR), and gross domestic product (GDP). The fluctuation in government spending is particularly crucial, serving as an important metric for assessing sustainable development [[43], [44], [45]]. The relationship between economic development and government spending is posited to be U-shaped, where initial increases in spending stimulate development, followed by a potential decline [46]. This aligns with the Keynesian perspective that views government spending as a causal factor in economic growth in the short term [47]. Furthermore, the consumer price index (CPI), which excludes institutional households, is used to provide a more precise measure of inflation. The CPI encompasses the cost of goods and services intended for final consumption by all resident households and is employed as an approach to assess the overall price evolution of a fixed spending model [48]. As a measure of socioeconomic progress and the standard of living, the CPI reveals the rate of inflation in the economy [48]. The nominal return, being the product of expected inflation and the real return, suggests that in the absence of any correlation between real return and inflation, stock returns should exhibit a positive correlation with inflation [49]. Gross domestic product (GDP) is a pivotal economic indicator, measuring the total value of all goods and services produced within a country's borders over a specific period, typically one year or a quarter. The GDP growth rate indicates the rate of change in the aggregate level of economic activity within an economy over a given period [50]. This metric is essential for understanding the overall health and performance of an economy.

**Table 1:** Variable Measurement

Variable	Measurement	Source
**Dependent variable**		
Stock Market Returns	Composite index (CI)	Central Bank of Ghana (BoG)
**Independent Variables**		
Fiscal Policy	Government Spending (GS)	WDI
	Gross Domestic Product (GDP)	WDI
	Tax Revenue (TR)	WDI
	Industrial Production Index (IPI)	WDI
Monetary Policy	Interest rate (INT)	WDI
	Exchange rate (EXC)	WDI
	Money supply (M2)	WDI
	Consumer price index (CPI)	WDI
**Control variables**		
Dependency Ratio	LogDER	WDI
Unemployment Rate	LogUER	WDI
Economic Growth	LogEG	WDI
Political Stability	LogPS	WDI
Economic Loss/Disaster	logELD	WDI

his study considers the money supply (M2), interest rate (INT), exchange rate (EXC), and consumer price index (CPI) as key interchangeable indicators of monetary policy. Monetary policy is posited to influence stock values through both direct and indirect channels. Directly, it affects the cost of capital and investment decisions, while indirectly, it impacts the level of uncertainty faced by economic agents, subsequently influencing variables that affect dividends and the stock return premium [50].

The money supply, an indicator of the economy's liquidity, is crucial for understanding financial market dynamics. A decrease in interest rates coupled with an expansion in the money supply can impact investor sentiment towards the stock market [51]. This relationship is further nuanced by the assertion that such monetary easing could lead to a decrease in stock prices, affecting trading volumes and overall stock values [52]. The interest rate represents the cost of borrowing or the return on investment, reflecting the compensation borrowers pay to lenders for the use of their funds. The exchange rate, defined as the value of one currency in terms of another, plays a pivotal role in international trade and investment.

Taxation, as a fiscal policy tool, can stimulate economic activity by providing incentives in areas such as manufacturing, tourism, investment, and agriculture [53]. These activities can contribute to balanced economic growth, with potential positive effects on stock returns. Changes in tax legislation, particularly corporate tax rates, directly impact corporate earnings. Tax reductions typically increase business profits, which may, in turn, elevate stock values. Corporate profits, particularly in the industrial sector, are linked to industrial production levels. Increased industrial production can lead to higher consumer demand for goods, resulting in improved sales and earnings for businesses in this sector. Consequently, higher profits can positively influence stock values, reinforcing the interconnectedness of fiscal and monetary policies with stock market performance.

#### 3.1.3 Control Variables

This study embarks on an exploratory quest to unravel the intricate dynamics governing economic events by establishing interconnections between various components. A critical aspect of this scientific pursuit is the identification and control of variables that could potentially affect or obscure the observed data. Adjusting for demographic shifts, political events, natural disasters, sectoral performance, global economic conditions, and national unemployment rates, as per the extant literature, significantly enhances the precision and reliability of the research findings.

Demographic changes, such as aging populations, can significantly influence fiscal policy, particularly in areas like healthcare and pension expenditures [54]. These trends may, over time, impact the sustainability of fiscal policies. Life-cycle changes in income, consumption, savings, investments, and wealth accumulation form the basis for research into the effects of aging on asset markets. A thorough understanding of these interactions is essential to prevent obsolescence of related policies. Political events have been widely recognized as key variables influencing a country's stock market [55]. Political instability can erode investor confidence and increase stock market volatility, casting doubt on the projected cash flows from investments [56]. Given that fiscal and monetary policies are shaped by political stability and regulatory frameworks, shifts in political ideology or leadership can precipitate changes in policy direction.

Natural disasters can induce substantial financial market volatility and economic loss, potentially affecting economic activity and financial asset volatility in other regions or countries [57]. The interconnectedness of global financial markets means that economies of countries with close ties to the affected nation also suffer repercussions. This interconnectedness is a product of increasingly open financial markets, advanced information systems, and maturing international capital markets. Research by [58] on the impact of natural catastrophes on financial markets indicates that increased government investment, trade openness, and per capita income can bolster the market's resilience to such adverse effects. The national unemployment rate serves as a barometer for the labor market and the broader economy. Elevated unemployment may signal underlying financial difficulties. Table 1 illustrates the potential impact of global economic trends on domestic stock market performance, highlighting the interplay between local market dynamics and worldwide economic conditions.

Given the multifaceted nature of economic systems, there is a pressing need for research that accounts for a myriad of exogenous factors influencing fiscal and monetary policies, as well as stock market performance. Understanding these complexities is vital for crafting effective policy responses and investment strategies in an increasingly globalized and interdependent world. By integrating a wide array of variables into economic models, researchers and policymakers can better anticipate challenges, seize opportunities, and foster sustainable economic growth and stability.

### 3.2 Model Specification

This study employs Autoregressive Distributed Lag (ARDL) models to assess the relationships between independent and dependent variables. ARDL models are particularly advantageous in the context of time-series analysis, as they are well-equipped to examine both short-term and long-term dynamics [[59], [60]]. The choice of ARDL models for this time-series study is strategic, as they facilitate an exploration of the dataset's temporal fluctuations, offering insights into immediate and sustained effects [[61], [62]]. ARDL models provide a robust framework for analyzing the interdependencies within economic data, allowing for a nuanced understanding of how variables interact over time. The formulation of ARDL models typically incorporates lagged values of the variables, reflecting the influence of past observations on current states. This approach is crucial for capturing the complex temporal relationships inherent in economic time-series data as expressed in equation (1), (2), (3), (4):

(1) $${\Delta CI}_{t}=\alpha +{\sum_{\iota =1}^{n}\beta_{1}\Delta X}_{it}+{\sum_{j=1}^{m}\gamma_{j}\Delta Y}_{jt}+{\sum_{l=1}^{q}\theta_{1}\Delta Z}_{lt}+{\sum_{\iota =k}^{p}\delta_{1}\Delta \mathrm{CI}}_{t-k}+{\;\varepsilon }_{t}$$

Where ${\Delta CI}_{t}$ represents the first difference of the dependent variable Composite index (ASR) at time *t*. ${\Delta X}_{it}$ represents the first differences of the independent variables related to fiscal policy (Government Spending, Gross Domestic Product, Tax Revenue, Industrial Production Index) at time *t.* ${\Delta Y}_{jt}$ represents the first differences of the independent variables related to monetary policy (Interest Rate, Exchange Rate, Money Supply, Consumer Price Index) at time *t*. ${\Delta Z}_{lt}$ represents the first differences of the additional control variables (Dependency Ratio, Unemployment Rate, Economic Growth, Political Stability, Economic Loss/Disaster) at time *t.* ${\Delta CI}_{t-k\;}$ represents lagged values of the dependent variable ASR up to lag order *p*. $\beta_{1},\gamma_{j},\theta_{1},\delta_{1}$ are the coefficients of the respective variables.

The Error Correction Mechanism (ECM) equation is added to account for the long-term equilibrium relationship and is specified as in equation 2:

(2) $${\Delta CI}_{t}={\alpha \;}_{1}{ECM}_{t-1}+{\sum_{\iota =1}^{n}\beta_{1}\Delta X}_{it}+{\sum_{j=1}^{m}\gamma_{j}\Delta Y}_{jt}+{\sum_{l=1}^{q}\theta_{1}\Delta Z}_{lt}+{\sum_{\iota =k}^{p}\delta_{1}\Delta \mathrm{CI}}_{t-k}+{\;\varepsilon }_{t}$$

ECM_t−1_ is the lagged error correction term, which represents the process of adjusting toward the long-run equilibrium relationship. The study further adopted the Exponential Generalized Conditional Heteroscedasticity (EGARCH) to investigate the volatility of stock market returns in the Ghanaian market as a result of changes in fiscal and monetary policies. The EGARCH is a variant of the ARCH/GARCH model commonly used in financial econometrics to model the conditional variance of a time series, especially in the context of financial asset returns. It allows for asymmetric responses to positive and negative shocks, capturing the leverage effect often observed in economic data. The EGARCH model is defined in equation 3:

(3) $$\log\;\left(\sigma_{t}^{2}\right)=\omega +\sum_{\iota =1}^{p}\alpha_{i}\;\left(\left|r_{t-1}\right|-\sqrt{\frac{2}{\pi }}\right)+\sum_{i=1}^{q}\beta_{1}og\;\left(\sigma_{t-1}^{2}\right)+{\sum_{\iota =1}^{n}\beta_{1}\Delta X}_{it}+{\sum_{j=1}^{m}\delta_{1}\Delta Z}_{jt}$$

Where $\sigma_{t}^{2}$ is the conditional variance of CI at time *t.* $r_{t}$ is the return series at time *t.* ω *is* the constant term. $\alpha_{i}$ and $\beta_{i}$ are the coefficients of the EGARCH model capturing the volatility dynamics. *p and q* are the orders of the EGARCH terms. ${\Delta X}_{it}$ represents the fiscal and monetary policy independent variables (e.g., GS, GDP, TR, IPI, INT, EXC, M2, CPI) at time *t*. In this EGARCH model, we account for the conditional variance of ASR as a function of past squared returns and the logarithm of past conditional variances, along with the effects of fiscal and monetary policy variables. The EGARCH model captures the asymmetric response of volatility to shocks, allowing for a more comprehensive analysis of the volatility dynamics of CI.

To estimate the spillover effect of the interaction between fiscal and monetary policy on stock market returns using a Vector Autoregression (VAR) model, we extend the traditional VAR framework to include the interaction terms between fiscal and monetary policy variables. Here's how we can formulate the VAR model as seen in equation 4:

(4) $$Y_{t}={\alpha \;}_{1}+{\sum_{\iota =1}^{p}A_{1}Y}_{t-1}+{\sum_{i=1}^{q}\beta_{i}X}_{t-1}+{\sum_{i=1}^{r}C_{1}Z}_{t-1}+\sum_{\iota =1}^{s}D_{1}\left(X_{t-1}*Z_{t-1}\right)+{\;\varepsilon }_{t}$$

The interaction terms (X_t-1 ∗_ Z_t-1_) capture the joint effect of fiscal and monetary policy variables on stock market returns. By estimating the VAR model with these interaction terms, we can analyze how changes in fiscal policy interact with changes in monetary policy to affect stock market returns. Additionally, impulse response analysis and variance decomposition can be performed to understand further the spillover effects of these interactions on stock market dynamics.

## 4 Empirical Results and Discussions

**Fig. 3:**
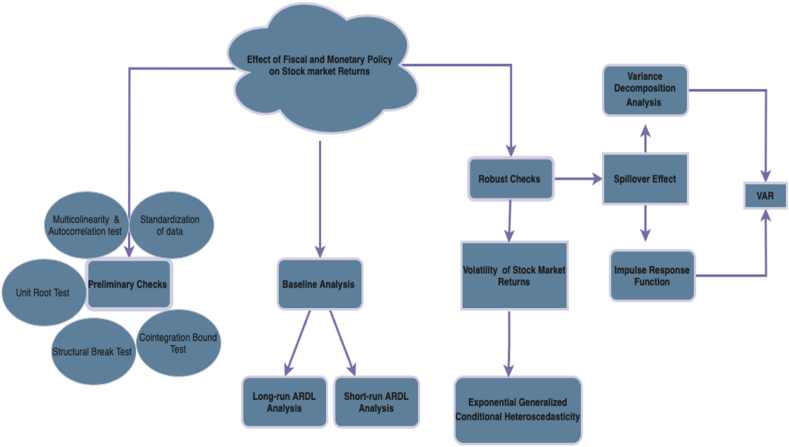
Roadmap of data analysis

There is considerable discourse and interest in the academic and policymaking communities regarding the correlation between monetary and fiscal policies and their influence on stock market performance. Fiscal policy, encompassing government decisions on borrowing, taxation, and spending, has the potential to affect investor sentiment, corporate profitability, and aggregate demand, all of which can significantly shape stock market dynamics. In parallel, monetary policy, which involves central bank actions such as interest rate adjustments and money supply management, can influence stock market performance by altering the cost of borrowing, liquidity availability, and economic outlook expectations. The complex and multifaceted relationship between these two policy levers and their impact on the stock market is crucial for macroeconomic stability, asset pricing, and investor behavior. It is imperative for policymakers, investors, and economists to have a nuanced understanding of how these policies interact and influence stock market dynamics in a rapidly changing global economy. This study, as detailed in this section and illustrated in Fig. 3, has conducted a series of preliminary tests to ensure the robustness of the analysis. These tests include baseline and robustness checks, as well as assessments for normality, multicollinearity, stationarity, and cointegration. These methodological rigors are essential to establish a reliable foundation for the empirical investigation of the interplay between monetary and fiscal policies and stock market behavior.

### 4.1 Preliminary Analysis

#### 4.1.1 Descriptive Statistics

Table 2 presents descriptive statistics for key variables pivotal to understanding the relationship between monetary and fiscal policies and stock market performance. The dependent variable, LogCI (Stock Market Performance), exhibits a mean of 17.497 and a standard deviation of 1.279, indicating a moderate central tendency with relatively low dispersion, which may imply a stable yet potentially volatile stock market trend for Ghana. Among the independent variables representing fiscal policy, Government Spending (LogGS) has a mean of 21.306 and a standard deviation of 0.913, while Gross Domestic Product (LogGDP) shows a higher mean of 23.721 and a standard deviation of 1.064. The Industrial Production Index (LogIPI) demonstrates a lower mean of 0.696 with a comparatively higher standard deviation of 2.271. Tax Revenue (LogTR) is characterized by a mean of 2.592 and the lowest standard deviation at 0.187, suggesting a high level of stability. The fiscal policy indicators, LogGS and LogGDP, exhibit moderate means and standard deviations, indicative of their significance and general stability within the economy. Turning to monetary policy metrics, the Interest Rate (LogINT) has a mean of 2.768 and a standard deviation of 0.425. The Exchange Rate (LogEXC) is relatively stable with a mean of 4.555 and a standard deviation of 0.206. The Money Supply (LogM2) shows greater variability with a mean of 22.001 and a standard deviation of 2.673. Lastly, the Consumer Price Index (LogCPI), a key measure of inflation, has a mean of 2.802 and a standard deviation of 0.600. The variability observed in the money supply (LogM2) suggests that it may be subject to more pronounced fluctuations compared to other monetary policy variables. These descriptive statistics provide a foundational understanding of the data distribution and are instrumental in subsequent analyses that explore the intricate dynamics between policy instruments and stock market outcomes.

**Table 2:** Descriptive Statistics

	Mean	Median	Max	Min	Std. Dev.	Skewness	Kurtosis	Jarque-Bera	Prob.	Sum	Sum Sq. Dev.
LogCI	17.497	17.599	19.474	15.226	1.279	-0.308	2.213	1.373	0.503	577.40	52.42
LogGS	21.306	21.318	22.787	20.043	0.913	0.132	1.463	3.341	0.188	703.09	26.71
LogGDP	23.721	23.935	25.094	22.329	1.064	-0.02	1.240	4.258	0.118	782.81	36.25
LogTR	2.592	2.576	3.079	2.268	0.187	0.994	3.785	6.290	0.043	85.56	1.12
LogIPI	0.696	-0.673	3.659	-3.506	2.271	0.291	1.510	3.518	0.172	22.97	165.11
LogINT	2.768	2.612	3.576	2.184	0.425	0.503	1.996	2.779	0.249	91.36	5.80
LogEXC	4.555	4.545	4.970	4.222	0.206	0.202	2.198	1.109	0.574	150.33	1.36
LogM2	22.001	22.193	25.988	17.117	2.673	-0.238	1.839	2.165	0.338	726.04	228.65
LogCPI	2.802	2.7401	4.085	1.583	0.600	0.305	2.401	1.006	0.604	92.47	11.53
LogDER	4.371	4.332	4.580	4.220	0.114	0.565	1.943	3.294	0.192	144.27	0.41
LogEG	1.100	1.124	1.795	0.292	0.335	-0.524	3.322	1.656	0.436	36.31	3.60
LogELD	3.127	3.190	3.190	3.000	0.090	-0.707	1.500	5.843	0.053	103.19	0.264
LogPS	-1.74	-1.609	-0.916	-2.302	0.455	0.076	2.040	1.297	0.522	-57.44	6.65
LogTS	0.953	0.875	2.116	0.131	0.510	0.730	3.543	3.338	0.188	31.46	8.33
LogUER	1.63	1.589	2.347	0.774	0.456	0.187	2.212	1.044	0.593	53.79	6.66

#### 4.1.2 Normality Test

Table 1 highlights the necessity of testing for normality in dataset evaluation. Skewness and kurtosis metrics offer preliminary indications of distribution normality, with values near zero suggesting a normal distribution. The Jarque-Bera test provides a statistical measure of normality, where p-values below the conventional threshold indicate significant deviations from a normal distribution [63,64]. This formal test is crucial for validating the skewness and kurtosis observations and for determining the appropriateness of parametric statistical methods in the analysis. Conducting normality tests is essential for ensuring the validity of statistical analyses that rely on parametric assumptions. These tests help researchers decide whether data transformations are needed or if non-parametric methods should be used, thereby safeguarding the reliability of the study's conclusions.

**Fig. 4:**
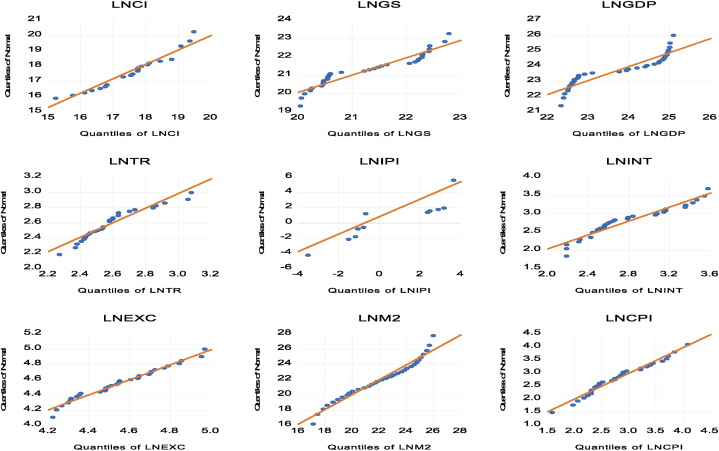
Normality of variables based upon Quantile-Quantile measure

Prior to conducting parametric analysis, the normality of the dataset was further estimated using a Q-Q plot, as depicted in Fig. 4. The quantile-quantile (Q-Q) plot is a graphical technique that compares the data distribution to a theoretical normal distribution. Each point on the Q-Q plot corresponds to a quantile of the dataset, plotted against the quantile of a normal distribution. A straight line in Fig. 4 would suggest that the data are approximately normally distributed, indicating the suitability of parametric statistical methods for the analysis.

#### 4.1.3 Multicollinearity and Autocorrelation Tests

The study employed autocorrelation and multicollinearity tests to ensure the reliability of the regression model. These diagnostics were conducted before presenting the results in Table 3. Table 3 presents a correlation matrix that delineates the relationships among variables representing monetary policy, fiscal policy, stock market performance, and control variables. The correlation matrix reveals significant positive associations between fiscal policy indicators such as government spending (logGS), the industrial production index (logIPI), and gross domestic product (logGDP), indicating the interconnectivity of fiscal activities. Additionally, substantial positive correlations exist between the money supply (logM2), government spending (logGS), gross domestic product (logGDP), and monetary policy, suggesting that monetary policy adjustments may be contingent upon current economic conditions.

**Table 3:** Correlation Matrix

	1	2	3	4	5	6	7	8	9	VIF
1.logCI	1									
2.logGDP	0.5811∗	1								3.2915
	0.0004									
3.logGS	0.6371∗	0.9119∗	1							2.4956
	0.0001	0.0000								
4.logTR	-0.0082	-0.2836	-0.2166	1						1.0529
	0.9637	0.1098	0.2259							
5logIPI	0.4617	0.7988∗	0.8520	-0.2761	1					3.6957
	0.0068	0.0000	0.0000	0.1199						
6.logINT	-0.5416∗	-0.6359	-0.7522	-0.1111	-0.5556	1				1.3087
	0.0011	0.0001	0.0000	0.5382	0.0008					
7.logEXC	-0.4646∗	-0.641∗	-0.7035	0.0035	-0.7688	0.4527	1			3.6448
	0.0064	0.0000	0.0000	0.9848	0.0000	0.0082				
8.logM2	0.6535∗	0.8482	0.9137	-0.0488	0.8220	-0.7093	-0.8366	1		2.7241
	0.0000	0.0000	0.0000	0.7876	0.0000	0.0000	0.0000			
9.logCPI	-0.2973	-0.3521	-0.4832	0.2334	-0.4458	0.6024	0.1748	-0.4472	1	2.5547
	0.0929	0.0445	0.0044	0.1911	0.0093	0.0002	0.3307	0.0091		
Farrar-Glauber Multicollinearity Chi^2^-Test										
Chi^2^ Test (P-Value) = 252.9348(0.4780)										
DW	2.5378									

Note: ∗∗∗, ∗∗, and ∗ denote significance at 1%, 5%, and 10% levels, respectively

Multicollinearity tests revealed significant correlations between predictors, which, while common, can complicate the interpretation of individual coefficients. However, the low levels of autocorrelation indicate that the model is not susceptible to residual serial correlation, thereby enhancing the robustness of the regression results. To mitigate multicollinearity, the variance inflation factor (VIF) was calculated for each variable. A VIF value exceeding 10 is typically indicative of multicollinearity concerns, yet all variables in this analysis exhibited VIF values below this threshold, suggesting no severe multicollinearity issues. Furthermore, the Farrar-Glauber Multicollinearity Chi2-Test yielded a non-significant p-value of 0.4780, providing further assurance of the absence of significant multicollinearity. The Durbin-Watson statistic (DW) of 2.5378 also supports the model's robustness, indicating minimal autocorrelation in the residuals. The regression analysis's resistance to multicollinearity and autocorrelation issues is confirmed by these diagnostics, validating the model's reliability. The non-significant VIF values and Durbin-Watson statistic, along with the Farrar-Glauber test's p-value, collectively affirm the regression results' credibility, ensuring that the conclusions drawn from the analysis are statistically sound

#### 4.1.4 Stationarity Test

Table 4 presents the outcomes of unit root tests conducted to assess the stationarity of the variables. The Phillips-Perron (PP), non-linear KPSS, and enhanced Dickey-Fuller (ADF) tests were employed to detect the presence of unit roots in both the levels and first differences of the variables. Variables that prove stationary in their first differences are classified as integrated of order one (I(1)), necessitating differencing to achieve stationarity.

**Table 4:** Unit Root Test

Variables	ADF		PP		Non-linear KPSS		Integration	Optimal Lag
	Level	1st Diff.	Level	1st Diff.	Level	1st Diff.		
logCI	-1.473	-4.148∗∗	-0.288-	-3.450∗∗	-0.119	-0.067∗∗	I (1)	1
logGDP	-2.028	-3.278∗∗∗	-3.056	-4.271∗∗∗	-0.103	-0.303∗∗	I (1)	1
logGS	-2.602	-2.028∗∗	-2.547	-3.729∗∗	-0.161	-0.632∗	I (1)	3
logTR	-1.041	-4.220∗∗	-1.847	-2.083∗∗	-0.11	-0.283∗∗	I (1)	4
logIPI	-2.606	-3.612∗∗	-2.745	-5.736∗∗	-0.104	-1.048∗∗∗	I (1)	4
logINT	-1.963	-7.280∗∗	-2.228	-6.321∗∗∗	-0.091	-0.295∗∗	I (0)	3
logEXC	-2.323	-4.122∗∗	-1.58	-3.367∗∗	-0.112	-0.137∗∗	I (1)	4
LogM2	-2.492	-2.381∗∗	-3.054	-4.263∗∗	-0.117	-0.253∗∗	I (1)	4
logCPI	-0.973	-3.204∗∗	-0.537	-1.820∗∗	-2.201	-0.318∗∗	I (1)	2
logDER	-0.118	-2.139∗∗∗	-0.585	-4.539∗∗∗	-2.269	-0.118∗∗	I (1)	2
logUER	-0.277	-5.281∗∗	-0.988	-4.783∗∗	-2.748	-0.328∗	I (1)	1
logTS	0.429	-1.405∗∗∗	-0.104	-4.209∗∗	-1.413	-1.379∗∗	I (0)	0
logEG	-1.963	-2.627∗∗	-0.292	-3.263∗∗∗	-0.306	-0.382∗∗	I (1)	1
logPS	-2.538	-3.482∗∗∗	0.415	-7.284∗∗	-0.456	-0.162∗	I (1)	3
logELD	-0.232	-4.418∗∗	-1.978	-3.572∗∗	-0.043	-0.381∗∗	I (1)	1

Note: ∗∗∗, ∗∗, and ∗ denote significance at 1%, 5%, and 10% levels, respectively

The selection of optimal lag lengths was informed by the Schwarz Bayesian Criterion (SBC) or the Akaike Information Criterion (AIC). Post-differentiation, the majority of variables exhibited stationarity, confirming their I(1) integration. Specifically, Stock Market Performance (logCI), Gross Domestic Product (logGDP), Government Spending (logGS), Tax Revenue (logTR), Industrial Production Index (logIPI), Exchange Rate (logEXC), Money Supply (logM2), Consumer Price Index (logCPI), Dependency Ratio (logDER), Unemployment Rate (logUER), Economic Growth (logEG), Political Stability (logPS), and Economic Loss/Disaster (logELD) demonstrated I(1) integration. In contrast, certain variables, such as the Technology Sector (logTS), indicated integration of order zero (I(0)), signifying that they are stationary at their level and do not necessitate differencing.

The unit root test findings are instrumental in confirming the order of integration for each variable, which is critical for the application of appropriate econometric models [65,66]. The identification of I (1) variables and the absence of severe multicollinearity or autocorrelation issues validate the analytical approach and the reliability of the subsequent econometric analysis.

Table 5 details the Zivot-Andrew test outcomes, which identify structural breaks in both levels and first differences of the variables, indicating shifts in their mean or trend. The test reveals significant structural breaks for most variables at various time points. For instance, the stock market performance (logCompositeindex) exhibited breaks in 1995 at the level and in 1999 in the first differences. The Gross Domestic Product (logGDP) showed breaks in 1999 at the level and in 2000 in the first differences. Tax revenue (logTR) had breaks in 2001 and 2005 in the first differences, while government spending (logGS) displayed breaks in 2000 at the level and in 1995 in the first differences. The presence of structural breaks in the time series data suggests that the underlying dynamics of the series have undergone changes at specific points in time. These breaks can have significant implications for the analysis, as they may affect the stability of models and the interpretation of results. The identification of such breaks is crucial for researchers and practitioners, as it may necessitate the use of models that account for structural changes or the segmentation of the data to analyze periods separately. This approach ensures that the analysis accurately reflects the economic phenomena under study and adjusts for any shifts in trends or volatility.

**Table 5:** Structural Breaks Unit Root Test

Variables	Zivot-Andrews test			
	At Level	Structural Break	At 1st Diff.	Structural Break
logCI	-3.208∗∗∗	1995	-1.272∗	1999
logGDP	-1.428∗∗	1999	-5.231∗∗	2000
logGS	-2.238∗	2000	-1.350∗∗∗	1995
logTR	-4.821∗∗	2005	-2.557∗∗∗	2001
logIPI	-1.328∗∗	1990	-4.588∗∗	2005
logINT	-5.207∗∗	1995	-3.138∗∗∗	1995
logEXC	-1.932∗∗	2010	-3.595∗∗	1999
LogM2	-3.285∗∗	2000	-4.318∗∗	2005
logCPI	-1.382∗∗	1996	-1.829∗∗	2006
logDER	-1.485∗∗	2011	-3.202∗∗	2010
logUER	-4.261∗∗	2005	-1.783∗∗	1990
logTS	4.014∗	2001	-2.172∗∗	2006
logEG	-6.025∗∗	1995	-4.559∗	1995
logPS	-4.290∗∗	2002	-5.604∗∗	2000
logELD	-2.488∗∗	1995	-3.292∗∗	2010

Note: ∗∗∗, ∗∗, and ∗ denote significance at 1%, 5%, and 10% levels, respectively

#### 4.1.5 Cointegration Test

Table 6 presents the results of cointegration bound tests for multiple models that explore the interactions among various variable sets. Model 1, which focuses on fiscal policies with a lag length of 4, reports an F-statistic of 4.7492. This value exceeds the lower bound of 2.589 (I(0)) and falls below the upper bound of 3.729 (I(1)), indicating cointegration between the variables lnGS, lnGDP, lnTR, and lnIPI at the 5% significance level. This finding provides strong evidence of a long-run equilibrium relationship between stock market performance and the fiscal policy indicators.

**Table 6:** Cointegration Bound Tests Analysis

F-Statistics	%	Lower Bound (I (0)	Upper Bound (I (1)	Lag length (p)
Fiscal Policies -**Model 1**		lnCI=f (lnGS, lnGDP, lnTR, lnIPI)		1,1,3,4,4
4.7492∗∗	5% significance	2.589	3.729	
Fiscal Policies with controls -**Model 2**		lnCI =f (lnGS, lnGDP, lnTR, lnIPI, lnDER)		1,1,3,4,4, 2,1,0
6.3822∗∗∗	10% significance			
Monetary Policies-**Model 3**		lnCI =f (lnINT, lnEXC, lnM2, lnCPI)		1,3,4,4,2
5.6082∗∗	5% significance			
Monetary Policies with controls-**Model 4**		lnCI =f (lnINT, lnEXC, lnM2, lnCPI, lnEG, lnPS, lnELD)		1,3,4,4,2,1,3,1
6.3929∗∗	5% significance			

∗, ∗∗, ∗∗∗, signifies 1%, 5%, and 10% significant respectively

Model 2 incorporates control variables by adding lnDER to the set of variables in Model 1. With a lag length of 4, Model 2's F-statistic of 6.3822 suggests cointegration between lnGS, lnGDP, lnTR, lnIPI, and lnDER at the 10% significance level. This extension strengthens the model by accounting for additional factors that may influence the relationship between fiscal policies and stock market performance.

Model 3, which examines the cointegration of monetary policy variables, reports an F-statistic of 5.6082, indicating a significant linkage between lnINT, lnEXC, lnM2, and lnCPI at the 5% level with a lag length of 4. This result falls between the critical values of 2.589 (I(0)) and 3.729 (I(1)), confirming the presence of a long-run equilibrium among these monetary policy indicators.

Model 4 expands upon Model 3 by incorporating additional control variables: lnEG, lnPS, and lnELD. The F-statistic for Model 4 is 6.3929, significant at the 5% level, demonstrating cointegration between the set of variables including the monetary policy indicators and the new control variables, all with a lag length of 4.

The cointegration results imply that there are stable long-term relationships among the variables of interest. This insight is valuable for understanding how fiscal policies, in conjunction with control variables, may influence stock market dynamics over time. The identification of cointegration also supports the use of Vector Error Correction Models (VECM) for further analysis, allowing for the examination of both short-term dynamics and long-term equilibrium relationships. The cointegration results for monetary policy variables suggest that there are stable, long-term relationships between interest rates, exchange rates, money supply, and the consumer price index. These findings imply that both monetary and fiscal policy actions can exert significant and enduring influences on stock market dynamics.

### 4.2 Main Results and Discussions

#### 4.2.1 Long-run Effect of Fiscal and Monetary policies on Stock Market performance

Understanding the enduring effects of fiscal and monetary policies on stock market performance is essential for thorough economic analysis. Fiscal policy assessments require consideration of a broad spectrum of economic variables and government interventions, including spending, taxation, and borrowing. Similarly, evaluating the long-term impact of monetary policy involves analyzing key factors such as interest rates, money supply, exchange rates, and the consumer price index (CPI), which collectively shape the economic environment and influence stock market dynamics.This study employs various models to dissect these relationships. The findings indicate that fiscal policies have mixed effects on stock markets, while monetary policies, especially interest rates, significantly influence market dynamics.

Table 7 encapsulates the long-run effects of fiscal and monetary policies on stock market performance. Model 1 reveals mixed effects of fiscal policies, with government spending (logGS) showing a positive but insignificant association, and tax revenue (logTR) exerting a significant positive impact. The coefficients for gross domestic product (logGDP) and industrial production index (logIPI) are negative, albeit not significantly so. Model 2 incorporates control variables, demonstrating a substantial positive relationship between tax revenue (logTR) and stock market performance, and a significant positive association with the dependency ratio (logDER), while the unemployment rate (logUER) shows no effect.

**Table 7:** Long run results

Variable	Model 1 Coefficient(t-stats)	Model 2 Coefficient(t-stats)	Model 3 Coefficient(t-stats)	Model 4 Coefficient(t-stats)
	Fiscal Policies on Stocks		Monetary Policies on Stocks	
The dependent variable is logCompositeIndex				
_Cons	1.065666 (-0.21)	-8.8705852 (-30)	19.03842 (1.49)	-6.78713 (0.33)
logCI	.0322447 (0.14)	.0475632 (0.19) ∗	-.102478 (-0.43)	-.1062759 (-0.38)
logGDP	-.2962247 (-0.60) ∗∗∗	-.2152766 (-0.59) ∗∗∗		
logGS	.8487729 (1.29)	.2305983 (0.29)		
logTR	1.968462 ∗∗ (1.89)	1.938394 (1.68) ∗∗		
logIPI	-.0305462 ∗ (-0.21)	.1420803 (0.76)		
logDER		4.99499 (0.93) ∗∗		
logUER		-.0623561 (-0.09)		
logTS		.3594572 (0.67) ∗∗∗		
logINT			-1.683303 (-2.35) ∗∗∗	-1.162531 (-1.44)
logEXC			.8558454 (0.44) ∗∗	1.475568 (0.66)
LogM2			.0507722 (0.23) ∗∗∗	.2488412 (0.98) ∗∗
logCPI			.3327994 (0.74)	.2345316 (0.51)
logEG				-.3894161 (-0.42) ∗∗
logPS				-1.349626 (-2.46)
logELD				5.052502 (1.29) ∗∗∗

∗, ∗∗, ∗∗∗, signifies 1%, 5%, and 10% significant respectively

For monetary policies, Model 3 shows a considerable negative impact of interest rates (logINT) on stock market performance, indicating the market's sensitivity to monetary policy adjustments. Exchange rates (logEXC) and money supply (logM2) exhibit positive but insignificant connections, and the consumer price index (logCPI) does not significantly affect the market. Model 4, with additional control variables, reaffirms the negative influence of interest rates (logINT). Economic growth (logEG) and political stability (logPS) show minor negative correlations, whereas financial loss or disaster (logELD) has a significant positive relationship.

#### 4.2.2 Short-run Effect of Fiscal and Monetary policies on Stock Market performance

Table 8 presents the short-run effects of fiscal and monetary policies on stock market performance, with Model 1 focusing on fiscal measures. The model shows inconsistent results, with changes in GDP (ΔGDP) having a non-significant impact. An increase in government spending (ΔGS) is positively correlated with stock market performance, suggesting that it may provide an initial boost. However, changes in tax revenue (ΔTR) have a non-significant negative coefficient, indicating a minor negative influence. The industrial production index (ΔIPI) demonstrates a strong positive relationship, implying that higher industrial output is associated with increased stock market activity. Model 2, which includes control variables such as the unemployment rate (ΔUER) and dependency ratio (ΔDER), indicates limited short-term direct impacts on stock market performance due to non-significant coefficients.

**Table 8:** Short Run Results

Variable	Model 1 Coefficient(t-stats)	Model 2 Coefficient(t-stats)	Model 3 Coefficient(t-stats)	Model 4 Coefficient(t-stats)
	Fiscal Policies on Stocks		Monetary Policies on Stocks	
The dependent variable is logCompositeIndex				
ΔGDP	-.1221876 (0.14) ∗∗	-.1687313 (-0.59) ∗∗∗		
ΔGS	1.176237 (1.61) ∗∗∗	1.45718 (1.75)		
ΔTR	-1.146139 (-0.95)	-.4644079 (-0.34) ∗∗∗		
ΔIPI	.2037649 (1.15) ∗∗∗	.1590476 (0.81)		
ΔDER		1.121819 (1.41) ∗∗		
ΔUER		.2695004 (0.38)		
ΔTS		.2843385 (0.67) ∗∗∗		
ΔINT			.0853007 (0.08) ∗∗∗	1.025527 (0.72) ∗∗∗
ΔEXC			-.1282657 (-0.06)	-2.045536 (-0.91)
ΔM2			-2.465652 (-0.81) ∗∗	-2.695537 (-0.63) ∗
ΔCPI			-.1229844 (-0.32) ∗∗∗	-.2996578 (-0.65) ∗∗∗
ΔEG				-.1886422 (-0.36)
ΔPS				--.7509079 (-1.60) ∗∗∗
ΔELD				10.75444 (1.66)
ECM (-1)	-.6382981 (-1.43) ∗∗	-.20486201 (-.93) ∗∗	-.4289204 (0.56) ∗∗	-.20373821 (1.83) ∗∗∗
Adjusted R^2^	0.538	0.593	0.628	0.713
SE	0.017	0.035	0.103	0.063
DW	2.232	2.429	2.217	2.058

∗, ∗∗, ∗∗∗, signifies 1%, 5%, and 10% significant respectively

Model 3 examines the short-term impact of monetary policy, finding that interest rates (ΔINT) have a non-significant coefficient, suggesting little direct effect on stock market dynamics. Exchange rates (ΔEXC) and money supply (ΔM2) also exhibit non-significant negative coefficients, indicating minimal short-term influence. The Consumer Price Index (ΔCPI) in Model 4 shows a non-significant negative coefficient, suggesting no substantial short-term impact on stock market performance. Political stability (ΔPS) and economic growth (ΔEG) have non-significant coefficients, implying no direct short-term effect. However, changes in the elderly dependency ratio (ΔELD) have a significant positive coefficient, suggesting that short-term fluctuations in the senior population proportion could influence stock market activity.

The Error Correction Model (ECM) coefficients at lag (-1) are instrumental in gauging the rate at which the system adjusts towards long-term equilibrium. In all models under review, significant ECM (-1) coefficients suggest a robust short-term correction mechanism, effectively steering the variables back to equilibrium following deviations. This finding underscores the models' ability to capture the dynamics of adjustment in response to short-term disequilibria. The adjusted R-squared values serve as a measure of the models' explanatory power, indicating the proportion of variance in stock market performance accounted for by the models. Higher values of the adjusted R^2^ indicate a better fit, reflecting a more comprehensive explanation of the data. The models' performance in explaining stock market variance is thus evaluated based on these values. Regarding diagnostic tests, Durbin-Watson (DW) values ranging from 2.058 to 2.429 suggest the presence of potential autocorrelation in the models. This observation warrants further investigation to refine the models and address any serial correlation issues, thereby enhancing their reliability and validity.

### 4.3 Robust Checks

#### 4.3.1 Robust Checks

Table 9 reports the outcomes of diagnostic tests that evaluate the robustness of the econometric model. The Breusch-Godfrey LM test, which is designed to detect autocorrelation in the residuals, yields a p-value of 0.8369. This result indicates that there is no evidence of autocorrelation, suggesting that the model's residuals are appropriately specified. The Breusch-Pagan-Godfrey test, used to assess heteroscedasticity, returns a p-value of 0.3429. This implies that the variance of the residuals is consistent across different levels of the independent variables, indicating no presence of heteroscedasticity. Additionally, the ARCH test, which examines the presence of autoregressive conditional heteroscedasticity (ARCH) effects, provides a p-value of 0.1832. This outcome suggests that there is no significant evidence of ARCH effects in the model, further confirming the stability of the model's error structure.

**Table 9:** Diagnostic Test of the Model

Items	Test	p-value	Results
Serial Correlation	Breusch Godfrey LM	0.8369	No autocorrelation issues
Heteroscedasticity	Breusch-Pagan-Godfrey	0.3429	No heteroscedasticity issues
	ARCH	0.1832	
Normality	Jarque-Bera Test	0.3027	Normal estimated residuals
Functional Form	Ramsey RESET Test	0.2739	Semantic model
Structural Stability	CUSUM	Stable	Model is Stable
	CUSUMsq	Stable	

**Fig. 5:**
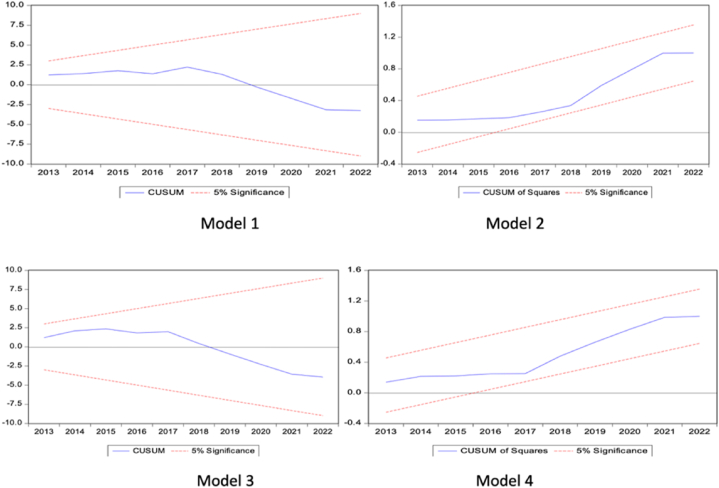
Stability Test

The Jarque-Bera test for normality of the residuals, with a p-value of 0.3027, supports the hypothesis that the residuals are normally distributed. This finding is crucial for the assumptions of many parametric statistical models. The Ramsey RESET test for the correct specification of the model's functional form yields a p-value of 0.2739, indicating that the model adequately fits the data without omitted variables or misspecification. As shown in Fig. 5, the CUSUM and CUSUMsq tests for structural stability provide consistent results, evidencing the model's long-term stability. These tests are vital for ensuring that the model remains valid over time and is not subject to structural changes that could affect its predictive power.

The positive results from the diagnostic tests, including the absence of autocorrelation, heteroscedasticity, ARCH effects, and the normality of predicted residuals, suggest that the model exhibits good statistical properties. These properties enhance the reliability of the model's results and affirm its validity for analyzing the impact of monetary and fiscal policies on stock market performance. The structural stability tests and the Ramsey RESET test further validate the model's suitability for longitudinal analysis. Collectively, these findings indicate that the model provides a robust analytical framework for examining the dynamics of the stock market in response to economic policies.

Table 10 presents the results of the Granger Causality Test, which assesses the causal relationships between economic variables. Specifically, the test delineates the causal links between fiscal and monetary policies and stock market performance. The findings reveal a bidirectional causality within fiscal policy, where changes in GDP (dGDP) and tax revenue (dTR) are reciprocally linked with stock market performance (dCompositeindex). This suggests that movements in the stock market can both influence and be influenced by fiscal indicators. Conversely, monetary policy exhibits unidirectional causal relationships with the stock market. The industrial production index (dIPI), consumer price index (dCPI), money supply (dM2), exchange rates (dEXC), and interest rates (dINT) are found to have one-way causal effects on stock market performance. These results highlight the directional influence of monetary policy variables on the stock market, indicating that policy adjustments in these areas can lead to changes in stock market dynamics.

**Table 10:** Granger Causality Test

Causality	F-Statistic	p-value	Decision
dGDP⇾ dCI	0.32856	0.009	Bi-directional relationship
dCI ⇾ dGDP	6.33582	0.007	
dGS⇾ dCI	0.54378	0.580	Uni-directional relationship
dCI ⇾dGS	0.11597	0.003	
dTR⇾ dCI	1.10017	0.008	Bi-directional relationship
dCI ⇾dTR	0.48292	0.004	
dIPI⇾ dCI	0.16337	0.001	Uni-directional relationship
dCI ⇾dIPI	0.86429	0.012	
dCPI⇾ dCI	0.42401	0.009	Bi-directional relationship
dCI ⇾ dCPI	1.64761	0.004	
dM2⇾ dCI	0.45451	0.637	Uni-directional relationship
dCI ⇾dM2	1.10868	0.001	
dEXC⇾ dCI	0.29305	0.004	Uni-directional relationship
dCI ⇾dEXC	0.13378	0.840	
dINT⇾ dCI	2.08190	0.050	Uni-directional relationship
dCI ⇾dINT	0.05226	0.922	

### 4.4 Further Analysis

#### 4.4.1 Changes in fiscal and monetary policies impact stock market volatility and returns

**Fig. 6:**
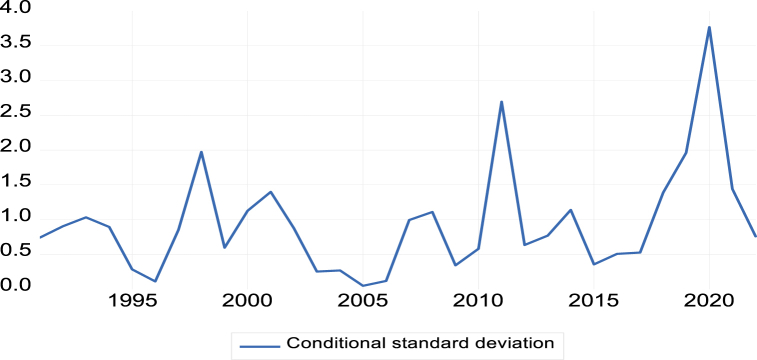
Stock market volatility based upon changes in fiscal and monetary policies

The study employs the Exponential Generalized Autoregressive Conditional Heteroskedasticity (E-GARCH) model to delve into the effects of fiscal and monetary policy changes on stock market volatility and returns. This sophisticated technique allows for an in-depth analysis of the dynamic relationship between policy adjustments and their impact on market volatility and returns, providing valuable insights into the complex interplay between economic policies and stock market behavior. The E-GARCH model, as depicted in Fig. 6, is particularly adept at capturing the conditional volatility of stock market returns, a feature that is crucial for understanding market dynamics. Known for its effectiveness in detecting volatility clustering in financial time series, the E-GARCH model is utilized to assess how changes in fiscal and monetary policies influence the levels of stock market volatility over different time periods.

By leveraging the E-GARCH modeling approach, the study enhances the understanding of the intricate linkages between fiscal and monetary policies and their implications for stock market dynamics. The graphical representation of conditional volatility from the E-GARCH model offers a visual insight into the potential short-term and long-term effects of policy changes on market stability and investor returns. The findings from this analysis contribute to the literature by highlighting the importance of considering policy-induced volatility when evaluating the impact of economic policies on financial markets. The E-GARCH model serves as a robust tool for researchers and policymakers seeking to understand and predict the market's response to policy interventions.

The E-GARCH model results, detailed in Table 10, reveal the effects of monetary and fiscal policies on stock returns. In the fiscal policy model, GDP exerts a significantly negative influence on stock returns, with a coefficient of -0.0254899 (t-statistic = 5.29, p < 0.01). Conversely, government spending (GS), tax revenue (TR), and the industrial production index (IPI) exhibit significant positive effects on stock returns, with respective coefficients of 0.143792 (t-statistic = 6.29, p < 0.01), 0.248934 (t-statistic = 3.08, p < 0.01), and 0.174392 (t-statistic = 3.95, p < 0.01). In the monetary policy model, interest rates (INT) significantly negatively affect stock returns, with a coefficient of -0.137328 (t-statistic = -6.22, p < 0.01). Exchange rates (EXC) and money supply (M2) display significant positive effects, with coefficients of 0.180208 (t-statistic = 5.63, p < 0.05) and 0.120919 (t-statistic = 5.45, p < 0.01), respectively. Both models feature a substantial constant term, indicating a baseline effect on stock returns. The adjusted R-squared value of 0.6932 for the models indicates that the independent variables explain approximately 69.32% of the variance in stock returns. The robustness of the models is further supported by diagnostic tests, including the ARCH-LM Test and the Ljung-Box Test, which do not detect any residual autocorrelation or ARCH effects.

The EGARCH model findings contribute to the understanding of how fiscal and monetary policies influence stock market returns. The significant coefficients and diagnostic test results validate the models' reliability and suggest that they provide a robust framework for analyzing the complex dynamics between economic policies and stock market performance.

#### 4.4.2 Spillover effects between fiscal and monetary policy measures on the stock returns (VAR)

The Vector Autoregression (VAR) methodology is employed to investigate the spillover effects between monetary and fiscal policy measures on stock returns. This approach allows for the examination of how shocks in one policy variable influence others and subsequently stock returns by estimating a system of equations. VAR elucidates the dynamic interrelationships between variables over time, offering insights into the mechanisms and extent of policy impacts on the stock market.

**Fig. 7:**
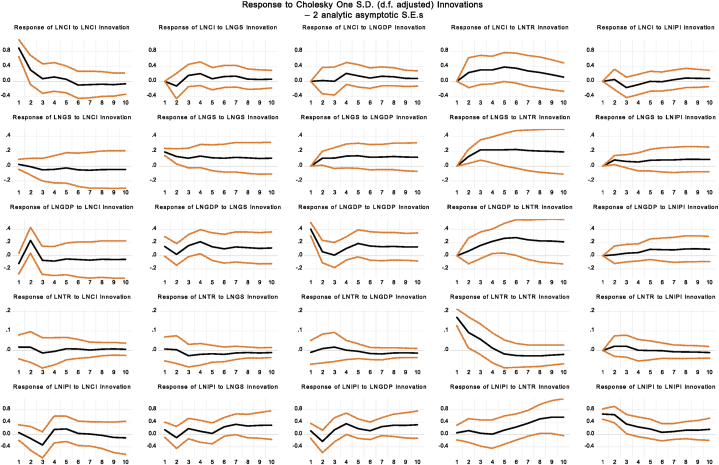
Impulse Response Function based upon Fiscal Policies

VAR analysis is particularly valuable for revealing the intricate connections between government actions and stock market behavior within the fiscal and monetary policy realms. It provides a comprehensive view of the direct and indirect effects of policy changes, such as government spending, taxation, interest rates, and money supply, on stock returns. For example, increased government spending under expansionary fiscal policy can boost business profits and stock returns, while contractionary monetary policy with higher interest rates might negatively affect market outlook and returns. The impulse response functions, as illustrated in Fig. 7, offer a dynamic view of how stock returns react to shocks from fiscal policy initiatives. The graph demonstrates the response of stock returns to one-unit shocks in key fiscal variables like GDP, tax revenue (TR), government expenditure (GS), and the industrial production index (IPI) across various forecast horizons.

The composite index response to stock return shocks shows an immediate positive reaction, indicating a temporary increase in market volatility, which then dissipates over time as the market stabilizes. The GDP response initially negative suggests a short-term decline in economic production following market disruptions, followed by a positive trend that points to a recovery in economic activity. The response of government spending (GS) to stock return shocks is positive, indicating an immediate increase in public expenditure, which has a lasting effect, reflecting a sustained fiscal stimulus. Tax revenue (TR) also responds positively to stock return shocks, with an immediate increase in tax receipts that gradually diminishes over time. The industrial production index (IPI) shows varied responses to stock return shocks. An initial positive response indicates a temporary rise in industrial output, with the effect fluctuating over time as depicted in Fig. 7. The VAR analysis and impulse response functions provide a robust framework for understanding the complex dynamics between policy changes and stock market performance. These tools reveal the short-term and long-term effects of fiscal policy shocks on economic variables and stock returns, contributing to a more nuanced understanding of policy impacts in financial markets.

**Fig. 8:**
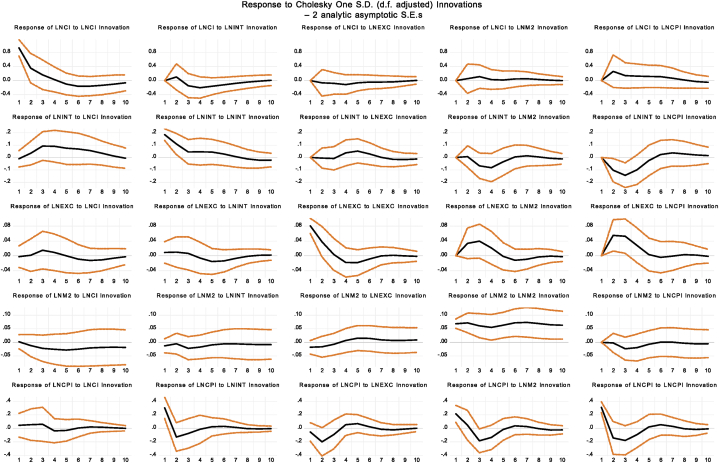
Impulse Response Function based upon Monetary Policies

Fig. 8 presents the impulse response function based on monetary policies, detailing the dynamic reactions of stock returns to shocks from various monetary policy interventions. The figure illustrates the response of stock returns to one-unit shocks in key monetary factors such as the money supply (M2), consumer price index (CPI), interest rates (INT), and exchange rates (EXC) over different forecast horizons.

Upon examination of the impulse response function, distinct patterns emerge in the reaction of stock returns to different monetary policy shocks. Initially, stock return shocks lead to an immediate increase in market volatility, indicating a spike in volatility that gradually subsides over time, suggesting a return to equilibrium in stock market performance. For interest rates, the initial shock has a minimal effect on stock returns, but a positive response is observed in subsequent periods. This indicates a delayed reaction to interest rate adjustments, suggesting that the full impact of interest rate changes on stock returns may not be felt immediately.

Exchange rate shocks also exhibit a minimal immediate effect on stock returns. However, over time, stock returns show a positive response, implying a delayed impact of exchange rate fluctuations on the stock market. The money supply shocks initially have a slightly positive impact on stock returns, but this effect diminishes in later periods, indicating a temporary increase in stock returns followed by a decline. In contrast, shocks to the consumer price index (CPI) elicit a positive initial reaction in stock returns, suggesting a short-lived increase in market volatility. Over time, as shown in Fig. 8, this effect inverts, with stock returns exhibiting a negative response in later periods.

Table 11 provides insights from a variance decomposition analysis focusing on fiscal policies and their influence on stock returns. The analysis reveals intriguing trends in the contribution of various fiscal variables to stock return volatility. In the initial forecast period, the variance in stock returns (dCOMPOSITEINDEX) is entirely attributed to its own shocks, demonstrating complete self-explanatory power. However, this proportion declines over time. Specifically, stock returns account for 100% of their own variation in the first period, but this share reduces to 65.47% by the fourth period, suggesting a diminishing self-influence over successive periods. Panel B illustrates the response of GDP (dGDP) to stock return shocks, indicating that the contribution of GDP to its own volatility increases over time. This trend suggests that as the forecast period extends, GDP plays a more significant role in explaining its fluctuations.

**Table 11:** Variance Decomposition Analysis based upon Fiscal Policies

Forecast	Impulse Variables					
Period	S.E.	dCI	dGDP	dGS	dTR	dIPI
Panel A	Response on dCI					
1	0.889032	100.0000	0.000000	0.000000	0.000000	0.000000
2	0.977587	91.36265	0.003207	0.442728	8.087814	0.103605
3	1.055292	78.49098	0.683264	3.194524	15.26201	2.369216
4	1.158346	65.47385	7.236941	4.156690	20.71226	2.420260
Response on dGDP						
Panel B	S.E.	dCI	dGDP	dGS	dTR	dIPI
1	0.450789	8.612517	91.38748	0.000000	0.000000	0.000000
2	0.517583	25.82306	71.31111	0.075692	2.748009	0.042132
3	0.582136	22.80205	57.90629	8.208295	10.67410	0.409254
4	0.687085	18.72595	49.33509	12.74572	18.24498	0.948254
Response on dGS						
Panel C	S.E.	dCI	dGDP	dGS	dTR	dIPI
1	0.189657	0.662771	9.260984	90.07624	0.000000	0.000000
2	0.309098	0.596822	26.77811	45.53088	20.81521	6.278976
3	0.425237	2.368535	26.57823	27.75407	37.64747	5.651698
4	0.526684	2.653904	29.56280	20.93597	41.81654	5.030782
	Response on dTR					
Panel D	S.E.	dCI	dGDP	dGS	dTR	dIPI
1	0.173946	1.771938	0.019347	0.756077	97.45264	0.000000
2	0.197950	2.786011	0.355297	0.603722	94.67290	1.582068
3	0.210067	2.494020	0.412648	3.441973	91.05965	2.591712
4	0.212181	2.553193	0.634009	4.898684	89.36940	2.544714
	Variance Decomposition of IPI					
Panel E	S.E.	dCI	dGDP	dGS	dTR	dIPI
1	0.710766	0.883450	5.582979	2.725239	0.530928	90.27740
2	1.007740	1.525650	8.115505	1.361346	1.740898	87.25660
3	1.138343	9.019965	9.362839	1.840065	1.369777	78.40735
4	1.233699	10.21288	15.96492	1.702116	1.166414	70.95367
Cholesky Ordering: COMPOSITEINDEX GDP GS TR IPI						

The variance decomposition for tax revenue (dTR) and government spending (dGS) is presented in Panels C and D, respectively. These panels show the varying contributions of these fiscal variables to stock return volatility across different forecast periods, highlighting their dynamic interplay with stock market performance. Finally, Panel E displays the variance decomposition for the industrial production index (dIPI). The results indicate that the index's contribution to the variation in stock returns diminishes over time, reflecting a weakening influence on stock market dynamics.

The variance decomposition analysis underscores the evolving impact of fiscal policy variables on stock returns. The initial dominance of stock returns in explaining their own volatility, coupled with the increasing influence of GDP and the varying contributions of tax revenue, government spending, and industrial production, provide a nuanced understanding of the fiscal policy effects on stock market behavior.

Table 12 presents the results of a variance decomposition analysis based on monetary policies. It shows exciting trends in how different monetary variables affect stock returns. Table 12 presents the variance decomposition analysis results based on monetary policies. In Panel A, stock returns (dCI) initially account for 100% of their variation, gradually decreasing over successive periods. Panel B illustrates the response of interest rate (dINT), with increasing contributions to its volatility over time. Panels C, D, and E respectively display the reactions of exchange rates (dEXC), money supply (dM2), and consumer price index (dCPI), showcasing their varying impacts on stock returns across forecast periods. Table 12 provides insights into the variance decomposition analysis based on monetary policies. Notably, the declining self-explanatory ability of stock returns (dCI) suggests increasing external influences over time. The growing contribution of interest rate (dINT) to its volatility implies its significant role in shaping stock market dynamics. Additionally, the varying impacts of exchange rates (dEXC), money supply (dM2), and consumer price index (dCPI) on stock returns underscore the complex interplay between monetary policies and market behavior. These findings have implications for policymakers and investors, highlighting the importance of monitoring and adapting to evolving economic conditions to mitigate risks and optimize investment strategies.

**Table 12:** Variance Decomposition Analysis based upon Monetary Policies

Forecast	Impulse Variable					
Period	Response on dCI					
Panel A	S.E.	dCI	d.INT	dEXC	dM2	dCPI
1	0.939425	100.0000	0.000000	0.000000	0.000000	0.000000
2	1.035071	94.72869	0.329769	0.267264	0.001329	4.672946
3	1.077167	89.22773	3.735518	0.747876	1.002963	5.285918
4	1.114025	83.77722	7.681610	1.912711	0.937695	5.690764
	Response on dINT					
Panel B	S.E.	dCI	d.INT	dEXC	dM2	dCPI
1	0.177611	0.002773	99.99723	0.000000	0.000000	0.000000
2	0.235531	3.248200	81.65986	0.149592	1.234534	13.70781
3	0.295243	12.48146	59.73308	0.317942	3.595207	23.87231
4	0.331729	13.53042	53.86328	1.721437	7.150571	23.73430
	Response on dEXC					
Panel C	S.E.	dCI	d.INT	dEXC	dM2	dCPI
						
						
1	0.083393	0.073023	1.416836	98.51014	0.000000	0.000000
2	0.106410	0.778803	1.020173	74.79817	5.289729	18.11312
3	0.123091	6.211191	1.846723	55.98555	10.90710	25.04943
4	0.132758	10.86669	3.329958	50.63973	10.69414	24.46948
	Response on dM2					
Panel D	S.E.	dCI	d.INT	dEXC	dM2	dCPI
1	0.073114	0.404295	6.764645	6.060046	86.77101	0.000000
2	0.104846	0.662818	4.763918	5.464716	89.05303	0.055515
3	0.128174	2.166694	6.730022	4.089012	84.52907	2.485202
4	0.143369	3.843411	6.876944	3.452760	82.81993	3.006952
	Response on dCPI					
Panel E	S.E.	dCI	d.INT	dEXC	dM2	dCPI
1	0.387167	7.287394	29.55503	1.401999	17.59158	44.16400
2	0.499920	7.490497	25.50756	20.21171	13.32820	33.46202
3	0.570667	9.544958	19.58182	19.21104	20.68320	30.97898
4	0.597927	11.24757	18.30300	18.69028	22.88691	28.87223
Cholesky Ordering: COMPOSITEINDEX INT EXC M2 CPI						

### 4.5 Further Discussions

The study provides valuable insights into the influence of fiscal and monetary policies on the performance of the Ghanaian stock market. By analyzing both the long-run and short-run effects of these policies, the research sheds light on the complex dynamics underlying stock market behavior in response to government interventions and monetary authority actions. The study delves into the theoretical foundations of fiscal and monetary policies' impacts on stock market returns, drawing upon seminal works in economics.

Tobin's model, a cornerstone in the field, underscores the profound impact of fiscal and monetary policy adjustments on stock returns, resonating with the complexities elucidated by [30]. Moreover, the study acknowledges the intricate interplay between government actions and future monetary policy decisions, aligning with previous research by [11,13] Theoretical frameworks further emphasize the multifaceted nature of the relationship between fiscal policy dynamics and stock market behavior, shedding light on their interactions.

In discussing fiscal policy's influence on stock market returns, the study elaborates on the Keynesian perspective, emphasizing the potential impact of budget deficits on interest rates and foreign investment [15]. Additionally, it touches upon the Ricardian equivalence theorem, delving into debates surrounding its plausibility and implications for stock market behavior [17]. Consistent with existing literature, the study's findings affirm the significance of fiscal policy variables such as government spending, the industrial production index, and tax revenue in shaping stock market performance [14,17,43].

Regarding monetary policy, the study explores various channels through which it affects stock market behavior, including the interest rate channel, portfolio balance channel, and credit channel [20]. Emphasizing the importance of understanding how monetary policy influences interbank markets and the real economy, the research aligns with prior studies that underscore the transmission mechanisms of monetary policy [25]. The empirical evidence provided by the study corroborates existing literature on the impact of interest rates, money supply, and exchange rates on stock market performance, reinforcing theoretical frameworks [[20], [21], [22], [23], [24], [25], [26]].

A comparative analysis of the study's findings with existing literature reveals both consistencies and divergences. While the study aligns with theoretical underpinnings and empirical evidence regarding fiscal policy's influence on stock market returns, such as the effects of government spending and tax policies, it introduces novel insights, particularly regarding the impact of the industrial production index. Similarly, the study's exploration of monetary policy channels and their effects on the stock market resonates with prior research, highlighting the significance of factors like interest rates and money supply. However, the study's findings on control variables such as economic growth and political stability add to existing literature, underscoring their pivotal role in shaping stock market dynamics.

## 5 Conclusions and Recommendations

### 5.1 Conclusion

The study provides a robust analysis of the intricate relationship between fiscal and monetary policies and their effects on the Ghanaian stock market. Key findings from both short-term and long-term impacts offer valuable insights into the complex dynamics that shape stock market behavior in response to policy changes. Through a synthesis of theoretical frameworks and empirical evidence, this research contributes to a deeper understanding of how government interventions and monetary authority actions influence stock market returns in Ghana. The results indicate that fiscal policy variables such as government spending, tax revenue, and the industrial production index have significant positive effects on stock returns, suggesting that expansionary fiscal policies may stimulate the stock market. Conversely, the negative impact of GDP changes on stock returns implies that economic growth slowdowns could be detrimental to market performance. Monetary policy instruments, particularly interest rates, were found to have a considerable negative impact on stock returns, highlighting the sensitivity of the stock market to monetary policy adjustments. The study also reveals that the money supply and exchange rates have positive, albeit delayed, effects on stock returns. This finding underscores the importance of considering the timing and sequencing of policy actions when formulating monetary policy. Additionally, the conditional volatility of stock market returns, as analyzed through the E-GARCH model, demonstrates the role of policy-induced volatility in market fluctuations.

### 5.2 Policy Recommendation

The study's findings reveal a positive correlation between government spending and tax revenue with stock returns. Therefore, it is advisable for fiscal policy to be strategically aligned with market conditions. Policymakers should carefully time and scale public investments and tax policies to invigorate the stock market, especially during economic downturns or periods of market lethargy. This proactive fiscal stance can serve as a counterbalance to market fluctuations, providing a stimulus when it is most needed.

The significant negative impact of interest rates on stock returns necessitates a prudent approach to monetary policy adjustments. Policymakers must consider the broader implications for investor confidence and market stability when formulating or revising interest rate policies. Clear communication and a gradual implementation of changes are essential to mitigate potential market disruptions. This measured approach can help maintain market equilibrium and investor trust.

The delayed but positive influence of money supply and exchange rates on stock returns underscores the importance of the sequencing of policy actions. Policymakers are encouraged to synchronize their interventions to maximize their impact on the stock market, considering the time lags inherent in market responses to policy changes. This strategic timing can lead to more effective policy outcomes and a more responsive stock market.

The application of sophisticated models like E-GARCH for analyzing market volatility underscores the necessity for advanced volatility forecasting capabilities. Policymakers should invest in enhancing their predictive and management skills concerning market volatility. The adoption of advanced modeling techniques can contribute to a more stable investment climate, reducing uncertainty and fostering investor confidence.

Understanding the intricate relationship between policy changes and stock market behavior necessitates improved investor education and transparent policy communication. Policymakers should actively engage with investors, elucidating the rationale for policy decisions and their anticipated market effects. This proactive communication can demystify policy intentions, build investor trust, and facilitate a more informed investment community.

Given the global interconnectedness of financial markets, policymakers must remain vigilant about global economic conditions and adjust domestic policies in response. This includes being agile in the face of international monetary policy shifts, trade developments, and geopolitical events that could affect the domestic stock market. A responsive policy framework can help shield the domestic market from adverse global influences and capitalize on emerging opportunities.

By integrating these refined recommendations, policymakers can more effectively harness fiscal and monetary policies to cultivate a stable, dynamic, and prosperous stock market. This approach not only supports the market during challenging times but also leverages policy tools to enhance overall economic health and investor prosperity.

### 5.3 Limitations

This study, while providing valuable insights, has certain limitations that should be acknowledged. Firstly, the findings are based on data specific to the Ghanaian stock market, which might not capture the broader dynamics of international financial systems. The Ghanaian stock market could be subject to unique regional factors, policy implementations, and investor behaviors that are not reflective of global trends or other emerging economies. Secondly, the study's focus on fiscal and monetary policies as determinants of stock market returns might not fully account for other influential factors such as technological disruptions, geopolitical events, or market-specific regulatory changes. Additionally, the empirical analysis is subject to the constraints of the data available, including potential issues with data quality, measurement errors, and the time frame of the data, which could affect the accuracy and robustness of the conclusions drawn.

### 5.4 Suggestions for Future Research

Given these limitations, there are several areas where future research could build upon the current study. Firstly, a longitudinal analysis could be conducted to examine the long-term effects of policy changes on the stock market, providing a more in-depth understanding of how these effects evolve over time. Secondly, a cross-country comparative study could help determine whether the observed relationships between fiscal and monetary policies and stock market performance are unique to the Ghanaian context or are more universally applicable. Thirdly, future studies could explore the differential impacts of specific policy instruments on various sectors of the stock market to identify which sectors are more responsive to particular policy changes. Additionally, the role of external factors such as global economic conditions and technological innovations in shaping the stock market's response to policy changes could be investigated. Lastly, employing alternative modeling techniques or expanding the current model to include more variables could capture additional complexities in the relationship between policy changes and stock market behavior.

## CRediT authorship contribution statement

**Benjamin Blandful Cobbinah:** Writing – review & editing, Writing – original draft, Methodology, Conceptualization. **Yang Wen:** Validation, Supervision, Investigation. **Francis Atta Sarpong:** Software, Formal analysis, Data curation.

## Data and code Availability

Data will be made available on request.

## Declaration of Competing Interest

The authors declare that they have no known competing financial interests or personal relationships that could have appeared to influence the work reported in this paper.
